# Investigating the Antidiabetic Effects of *Calotropis procera*: Preclinical Assessment

**DOI:** 10.1002/fsn3.71364

**Published:** 2026-08-05

**Authors:** Yu‐Cheng Kuo, Musa I. Ajibola, Halimat Y. Lukman, Bashir Lawal, Lung‐Ching Chen, Sheng‐Liang Huang, Olawale David Ajayi, Sunday Amos Onikanni, Saheed Sabiu, Leandro Miranda‐Alves, Hsu‐Shan Huang

**Affiliations:** ^1^ Department of Pharmacology, College of Medicine Taipei Medical University Taipei Taiwan; ^2^ College of Osteopathic Medicine Sam Houston State University Conroe USA; ^3^ Department of Psychiatry University of Maryland School of Medicine Baltimore Maryland USA; ^4^ Department of Biotechnology and Food Science, Faculty of Applied Sciences Durban University of Technology Durban South Africa; ^5^ UPMC Hillman Cancer Center, University of Pittsburgh USA; ^6^ Division of Cardiology, Department of Internal Medicine Shin Kong Wu ho‐Su Memorial Hospital Taipei Taiwan; ^7^ Graduate Institute of Aerospace and Undersea Medicine National Defense Medical University Taipei Taiwan; ^8^ Health Center Medical Laboratory Department Bamidele Olumilua University of Education, Science and Technology Ikere‐Ekiti Ekiti Nigeria; ^9^ Centro de Ciências da Saúde Instituto de Ciências Biomédicas Laboratório de Endocrinologia Experimental—LEEx Universidade Federal do Rio de Janeiro Rio de Janeiro Brazil; ^10^ Institute of Biomedical Sciences, Postgraduate Program in Pharmacology and Medicinal, Chemistry Federal University of Rio de Janeiro Rio de Janeiro Brazil; ^11^ Department of Chemical Sciences, Biochemistry Unit Afe‐Babalola University Ado‐Ekiti Ekiti Nigeria; ^12^ Graduate Institute of Cancer Biology and Drug Discovery College of Medical Science and Technology, Taipei Medical University Taipei Taiwan; ^13^ School of Pharmacy National Defense Medical University Taipei Taiwan; ^14^ Ph.D. Program in Biotechnology Research and Development College of Pharmacy, Taipei Medical University Taipei Taiwan

**Keywords:** *Calotropis procera*, hypoglycemia, inflammation, oxidative stress, therapeutics

## Abstract

Diabetes mellitus (DM) is a metabolic disease characterized by persistent hyperglycemia, oxidative stress, and inflammation, often leading to severe complications. Current antidiabetic drugs exhibit efficacy; however, they are limited by adverse effects and diminishing long‐term benefits. Natural products, including *Calotropis procera* , are promising alternatives with minimal side effects: This study investigates the antidiabetic and protective effects of the methanolic extract of *C. procera* (CMECP) against diabetes and its complications using in vitro and in vivo models. in vitro antioxidant, anti‐inflammatory, and hypoglycemic activities of the extract were conducted using established procedures while experimental rats were induced into diabetes with streptozotocin (STZ) and treated with distilled water, CMECP [150 and 300 mg/kg body weight (BW)] or 100 mg/kg b.wt metformin and assessed for their effects against diabetes and diabetes‐related complications. The extract exhibited dose‐dependent in vitro antioxidant, anti‐inflammatory, and hypoglycemic activities. Treatment with the extract resulted in a 65% decrease in the fasting blood glucose levels of the diabetic rats at 300 mg/kg BW after the treatment period. The extract also improved insulin secretion, reversed dyslipidemia, ameliorated alterations in liver and kidney function biomarkers, reduced oxidative stress markers, and enhanced antioxidant defenses in STZ‐induced diabetic rats. Inflammatory biomarkers such as cyclooxyegase‐2 and nuclear factor‐kappa B were suppressed, with improved hematological parameters and body weight. Additionally, CMECP mitigated diabetic‐induced neuronal cholinesterase activity without affecting neurotransmitter levels. The extract of *C. procera* demonstrated potent antidiabetic effects by modulating oxidative stress, inflammation, and biochemical dysfunctions in diabetes. These findings support its potential as a natural therapeutic agent for diabetes management. Further studies are ongoing to identify the constituents of the extract and elucidate their mechanisms of action for possible hit compounds identification for future antidiabetic drug development purposes.

## Graphical abstract

*Calotropis Procera* In vitro/Diabetic rodent model Improved β‐cell function, reduced hepatic gluconeogenesis antioxidant activity.

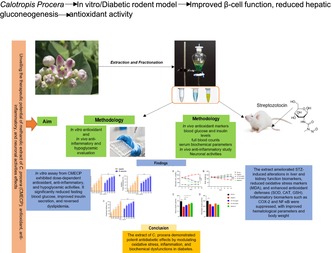

## 1 Introduction

Diabetes mellitus (DM) is a complex metabolic disease characterized by defective insulin secretion or resistance leading to persistent hyperglycemia (Petersmann et al. 2019), presenting symptoms such as excessive urination, sweating, dehydration, blurred vision, frequent tiredness, unintentional weight loss, and organ dysfunctions in severe or complicated cases (Ramachandran 2014; Mezil and Abed 2021). DM is classified into four: (i) Type 1 DM (T1DM) caused by deficient insulin secretion because of autoimmune destruction of pancreatic β‐cells; (ii) Type 2 DM (T2DM), which is known to be non‐insulin dependent; (iii) Gestational DM, which occurs during pregnancy and may disappear or progress after birth; and (iv) Juvenile DM, which occurs in children (Onikanni et al. 2021; ADA 2022). Type 2 diabetes mellitus (T2DM) is the most prevalent form of diabetes, representing 90% to 95% of all reported cases. It is more common in developed countries, primarily due to lifestyle factors, and poses a significant socio‐economic burden (Janssen et al. 2020; Malek et al. 2019). The disease ranked among the world's top 10 causes of death in 2024 (Zhou et al. 2024). Furthermore, an upsurge to over 1.31 billion by 2050 has been projected from the 529 million (6.1% of the world population) diabetic cases recorded in 2021 (Ong et al. 2023). Thus, there is an urgent need for development of more effective drugs to combat diabetes.

Oxidative stress and inflammation triggered by hyperglycemia and hyperlipidemia have been extensively implicated in the progression of T2DM and its complications. A hyperglycemic state results in free radical generation including reactive nitrogen species (RNS) and reactive oxygen species (ROS), glucose autooxidation, and endogenous antioxidants depletion, leading to oxidative stress, inflammation, and dysfunction of pancreatic β‐cells which results in decreased insulin secretion (Yaribeygi et al. 2020). Free radicals disrupt insulin signaling, leading to insulin resistance. High levels of oxidative stress activate NF‐κB, triggering the release of proinflammatory mediators that damage cells and impair their function. Studies in both animals and humans have shown that excessive ROS accumulation, NF‐κB activation, and resulting inflammation play a significant role in the development and progression of T2DM. This highlights the importance of inflammation in the pathogenesis and pathophysiology of T2DM (Suryavanshi and Kulkarni 2017; Tishkowski and Gupta 2025). The interplay between oxidative stress and inflammatory conditions contributes to the development of diabetic complications which include retinopathies, diabetic encephalopathy, nephropathies, coronary heart disease, and hypertension (Tomic et al. 2022). Therefore, therapeutic approaches targeting improvement of oxidative defense and reduction of inflammatory responses would be helpful in the management of DM and its complications.

Current antidiabetic medications are effective in managing diabetes by lowering glucose levels through increased insulin sensitivity or secretion. However, their use is limited due to loss of efficacy and severe side effects like acute hepatitis, diarrhea, skin sensitivity, and lactic acidosis (Onikanni et al. 2023; Arumugam et al. 2013; Thakare et al. 2017). Therefore, there is a need for development of alternative targeted therapeutic approaches that lower blood glucose and protect β‐cells from oxidative and inflammatory damage. Over the years, natural products, especially medicinal plants, have been shown to be effective and have minimal side effects when compared with conventional treatments based on evidence from traditional health practices and experimental studies (Onikanni et al. 2022). Similarly, preclinical studies have demonstrated potent antioxidant and anti‐inflammatory properties of plant‐derived extracts and compounds in the management of diabetes and its complications (Lukman et al. 2024; Abdulrasheed‐Adeleke et al. 2023; Yusuf et al. 2023). Therefore, exploring medicinal plant extracts and their bioactive metabolites may improve the prognosis of diabetes and its complications while modulating oxidative stress and inflammatory abnormalities and subsequently improve the morphological and physiological integrity of pancreatic β‐cells.

*Calotropis procera*, also known as Aiton, is a family of flowering perennial shrubs of the family Apocynaceae and subfamily Asclepiadaceae (the milkweed family), predominant in some Asian and African countries where it is used in traditional medicinal systems (Karale and Karale 2017). The plant is rich in phytoconstituents such as flavonoids, glycosides, triterpenoids, alkaloids, resins, anthocyanins, tannins, saponins, and proteolytic enzymes (Ahmad Nejhad et al. 2023; Shaker et al. 2010). It has been extensively demonstrated to possess activity against cancer, ulcer, bacterial infections, obesity, liver damage, inflammation, and oxidative stress (Dao et al. 2025). Although both in vitro (Yadav et al. 2023) and in vivo (Neto et al. 2013; Okwudiri Ihegboro et al. 2022) studies have demonstrated the antidiabetic activity of the plant, however, detailed investigation into the activity of *C. procera* against diabetes and related complications are limited. In this study, the effects of *C. procera* extract on glucose homeostasis and diabetes complications were investigated using in vitro and in vivo approaches. Our research aimed to investigate the effectiveness of *C. procera* extract in preclinical models of hyperglycemia and diabetes‐induced oxidative stress, inflammation, and neuronal impairment. This study may pave the way for utilizing *C. procera* extract as a potential alternative therapy for managing diabetes complications.

## 2 Materials and Methods

### 2.1 Experimental Animals

The experimental animals of Sprague Dowley rat [112.50 ± 7.39 g body weight (BW)] used in this study were obtained from Federal University of Technology, Minna (FUTMinna), Nigeria, animal holding unit. The animals were given free access to standard rat feed pellets and clean drinking water and maintained on a 12 h dark/light cycle.

### 2.2 Ethics Statement

Ethical approval was obtained from FUTMinna, Nigeria ethical committee and standard protocol for handling experimental animals was strictly adhered to. The study was in compliance with the guidelines on the operation of the Animals Act 1986 with ethical reference Number AE‐FUTMN‐2020/0099.

### 2.3 Chemicals, Kits, and Reagents

The inflammatory markers and neurotransmitter ELISA kits used in this study were purchased from Elabscience, United States while the insulin kit was from Calbiotech inc., El Cajon, California. The liver and kidney function assay kits used were products of Randox Laboratories Co‐Atrium, UK, commercial biochemical kits (Olympus, Hamburg, Germany) used on an automated analyzer (Olympus AU800). Other chemicals and reagents used in the study were of analytical grade and products of Sigma Aldrich Inc., St. Loius, USA.

### 2.4 Preparation of Extract

*Calotropis procera* plant was collected in June 2018, within Minna, Niger State, Nigeria, authenticated, and a specimen voucher was deposited prior to the commencement of the experiment. To prepare the extract, the plant sample was firstly cleansed to remove dust particles, air dried to a constant dry weight, and then pulverized into fine powder to increase the surface area for extraction. To 100 g of the pulverized sample, 500 mL of methanol was added and left for 3 days with occasional shaking for complete extraction. Afterwards, the crude methanolic extract of *C. procera* (CMECP) was obtained by concentrating the filtrate recovered from the mixture under reduced pressure. The extract was then stored for future biochemical assays.

### 2.5 In Vitro Studies

#### 2.5.1 Antioxidant Assays

The in vitro free radical scavenging ability of CMECP was analyzed using 2,2‐diphenyl‐1‐picrylhydrazyl (DPPH), ferric reducing antioxidant power (FRAP), and lipid peroxidation (LPO) assays. The DPPH assay was carried out by adding different concentrations of the extract (50–400 μg/mL) to 0.1 mL DPPH solution (obtained by dissolving 0.5 mL DPPH in 4.5 mL methanol) and incubated in the dark for 45 min. The absorbance was read against a blank at 517 nm (Tsado et al. 2016). The FRAP assay was carried out by adding 1 mL of different extract concentrations (50–400 μg/mL) to 1% potassium hexacyanoferrate (III) dissolved in 0.2 M sodium phosphate buffer. 10% trichloroacetic acid was then added, and the resulting mixture was incubated at 50°C for 20 min. The mixture was centrifuged to obtain a clear supernatant to which 0.1% ferric chloride was added, and the absorbance was read at 700 nm (Oyaizu 1986). The LPO assay was carried out using the thiobarbituric acid‐reactive substance (TBARS) method described by (Panjamurthy et al. 2005).

#### 2.5.2 Anti‐Inflammatory Assays

The human red blood cell membrane stability test was determined by centrifuging freshly collected human plasma at 3000 rpm for 10 min which was then washed with normal saline of the same volume. A 2 mL reaction volume was subsequently prepared with 10% of the red blood cell suspension and different concentrations of the CMECP and aspirin (reference standard). The mixture was incubated at 56°C for 30 min followed by centrifugation at 2500 rpm for 5 min and the absorbance was subsequently read at 560 nm (Thenmozhi et al. 1989). Protein denaturation assay was determined by mixing an aqueous solution of 1% BSA in different extract concentrations, heated at 55°C for 30 min. The mixture was allowed to cool, the turbidity measured, and the percentage inhibition of protein denaturation was calculated (Mizushima and Kobayashi 1968).

For proteinase inhibition assay, a 2 mL reaction mixture was made by mixing 0.06 mg trypsin, 1 mL Tris–HCl buffer and 1 mL extract (50–400 μg/mL). This was then incubated at 37°C for 5 min, after which 0.8% w/v casein was added and further incubated for 20 min. The reaction was terminated with perchloric acid (2 mL of 70%). A turbid suspension was obtained, centrifuged, and the absorbance of the supernatant read at 210 nm against Tris–HCl buffer as blank (Cirillo 1962).

#### 2.5.3 Hypoglycemic Assays

The percentage alpha‐amylase inhibition of CMECP and the reference standard (acarbose) was determined by incubating different concentrations of the extract (50–400 μg/mL) and acarbose (12.5–100 μg/mL) with porcine pancreatic amylase solution (prepared in 0.02 M sodium phosphate buffer (pH 6.9) at 37°C for 10 min). 500 μL of 1% starch solution (prepared in 0.02 M sodium phosphate buffer, pH 6.9) was subsequently added and further incubated at 37°C for 30 min. The reaction was terminated with 10 μL of 1 M HCl and color development done using iodine. The absorbance was then read at 580 nm and the percentage alpha‐amylase inhibition calculated (Hernández‐Montañez et al. 2012).

The amount of glucose taken up by yeast cells was carried out by adding different concentrations of glucose solution to different extract and standard concentrations followed by a 10 min incubation at 37°C. The reaction was initiated by adding 100 μL of yeast suspension (prepared by centrifuging commercial baker's yeast repeatedly at 3000 rpm for 5 min with distilled water till a clear supernatant was obtained and subsequently used to make a 10% v/v solution in distilled water), vortexed and incubated at 37°C for 60 min. The resulting mixture was centrifuged, and the amount of glucose taken up by the cell was measured in the supernatant (Lawal et al. 2022).

### 2.6 In Vivo Analysis

#### 2.6.1 Induction of Diabetes and Animal Grouping

The experimental animals were induced into diabetes via intraperitoneal administration of 40 mg/kg BW of streptozotocin (STZ) followed by 5% glucose solution administration post 24 h STZ injection. The fasting blood glucose (FBG) of the animals was measured and animals with 250 mg/dL sugar level were considered diabetic. The diabetic rats were randomized into five treatment groups (*n* = 5). Group 1 (normal control) comprised non‐diabetic rats and received 2 mL of distilled water, group 2 [diabetic untreated group (negative)] received 2 mL of distilled water, groups 3–5 were diabetic rats treated with 150, 300 mg/kg BW, and 100 mg/kg metformin, respectively. The choice of dosage was within the safe doses informed by previous study (Neto et al. 2013; Onikanni et al. 2022). All treatments were orally administered daily for 14 days.

#### 2.6.2 Collection and Preparation of Blood, Serum and Tissues Homogenate

After a 14‐day treatment period, the animals were fasted overnight, weighed, and sacrificed using diethyl ether anaesthetization. Blood was collected via the jugular vein and allowed to clot before centrifuging at 3000 rpm for 15 min; the serum was decanted and properly preserved in the refrigerator for biochemical analysis (Adewuyi et al. 2024; Onikanni et al. 2022). Blood samples used for hematological analysis were collected in EDTA bottles. The organs (liver, kidney, brain, and pancreas) were harvested, cleansed of blood, and weighed before homogenizing in 0.25 M cold sucrose solution. The resulting homogenates were centrifuged at 4000 rpm for 10 min (Oloyede et al. 2020). The supernatants obtained were then carefully preserved in the refrigerator for further biochemical analysis.

#### 2.6.3 Analysis of Blood Glucose and Insulin Levels

After successful induction of the rats into diabetes, the effect of CMECP on fasting blood glucose level of the diabetic rats was measured for 2 weeks at an interval of 3 days while insulin ELISA kit (catalog No: IN374S; Calbiotech inc., El Cajon, California) and ELISA reader (Sunrise, Tecan, Austria) were used to determine the insulin levels in the serum and pancreas.

#### 2.6.4 Analysis of Full Blood Counts

An automated hematologic analyzer (Sysmex, KX‐21, Japan) was used to analyze the hematological parameters such as erythrocyte indices [hemoglobin (HGB)], packed cell volume (PCV), red blood cells (RBC), mean‐corpuscular hemoglobin (MCH), mean cell volume (MCV), mean corpuscular hemoglobin concentration (MCHC), leucocyte indices such as white blood cells (WBC), and its differentials as well as thrombocyte index platelet (PLT) (Dacie and Lewis 1991).

#### 2.6.5 Analysis of Serum Biochemical Parameters

Biochemical parameters such as liver function, kidney function, lipid profiles, and electrolytes analyses were carried out in the serum to determine the effect of CMECP in diabetic‐induced complications using standard procedures. The LFT and KFT assays performed included alanine transaminase (ALT) and aspartate transaminase (AST) (Reitman and Frankel 1957), alkaline phosphatase (ALP) (Rec 1972), total protein (Gornall et al. 1949), bilirubin (Suzuki and Sakagishi 1994), albumin (Doumas et al. 1971), creatinine (Delanghe and Speeckaert 2011), urea (Searle 1984). Lipid profile assays included high density lipoprotein (HDL)‐cholesterol (Albers et al. 1978) and triglycerides (TGs) (Van Handel and Zilversmit 1957), total cholesterol (TC), all measured by colorimetric methods while the low‐density lipoprotein (LDL)‐cholesterol (mg/dL) was computed as [TC−(HDL + VLDL)] (Friedewald et al. 1972). The serum electrolyte concentration was analyzed as described by Tietz (1995).

#### 2.6.6 Analysis of Antioxidant Parameters

The method described by Misra and Fridovich (1972) was to estimate activities of superoxide dismutase (SOD). Briefly, to 0.2 mL of sample, 2.5 mL of 0.05 molL^−1^ of carbonate buffer (pH 10.2) was added. The reaction was initiated by the addition of freshly prepared 0.3 mmolL^−1^ epinephrine. The absorbance was read at 480 nm and changes in absorbance's were recorded every 30 s for 150 s to estimate the SOD activity. The activity of catalase (CAT) was estimated as described by Sinha (1972). To 0.1 mL of the serum or tissue supernatant, 1 mL of 0.01 M phosphate buffer of pH 7.0 and 0.4 mL of 0.2 M H_2_O_2_ solution were added. The resulting solution was gently mixed, and the reaction terminated by the addition of 2 mL dichromate acetic acid reagent. Reduced glutathione (GSH) levels were determined by a modified colorimetric protocol (Gl 1959). While lipid peroxidation biomarker; malondialdehyde (MDA) was assayed by TBARSs estimation (Shagirtha et al. 2017).

#### 2.6.7 Analysis of Inflammatory Biomarkers (COX‐2/NOx/Nf‐kb)

The levels of inflammatory biomarkers were analyzed using ELISA kits. For COX‐2, rat prostaglandin endoperoxide synthase 2 (PTGS2/COX‐2), catalog no E‐EL‐R0792 was used. NFKB activity was estimated using rat NFKB‐p105 Nuclear factor NF‐kappa‐B p105 subunit, catalog no, E‐EL‐R0673 which is based on the color development when Rat NFKB‐p105, is conjugated with Rat NFKB‐p105‐specific biotinylated detection antibody and Avidin‐HRP conjugate. The ability of nitric oxide (NOx) to reduce nitrate to nitrite was used to estimate nitric oxide (NOx) level according to the procedure reported by (Miranda et al. 2001).

#### 2.6.8 Analysis of Cholinesterase (CHEs)

Neuronal acetylcholinesterase (ACHE) and butyrylcholinesterase (BChE) activities were determined as described by (Ellman et al. 1961). The reaction mixture containing phosphate buffer (0.1 M, pH 8.0), DTNB (10 mM), 50 μL cytosol, and 150 mM of acetyl thiocholine iodide (ACHE assay) or 150 mM of butyryl thiocholine iodide (BChE assay) was incubated, and change in absorbances were monitored at 412 nm for 3 min. The serotonin assay was conducted using the ST/5‐HT (Serotonin/5‐Hydroxytryptamine) ELISA Kit (Catalog No: E‐EL‐0033, of Elabscience, United States) using the manufacturer protocols.

#### 2.6.9 In Vivo Anti‐Inflammatory Study Using Xylene‐Induced Ear Swelling Test

Xylene was used on the 14th day of treatment to induce ear swelling in all the treatment groups to determine the Erythrocyte Sedimentation Rate (ESR) and the effect on organ weight.

#### 2.6.10 Data Analysis

Experiment are conducted in independent replicate. Between‐group differences were assessed by one‐way analysis of variance (ANOVA) followed by Tukey’s honestly significant difference (HSD) post‐hoc test, with p < 0.05 considered statistically significant. All statistical analyses were performed using GraphPad Prism v8.0.

## 3 Results

### 3.1 *Calotropis procera* Extract Exhibited a Concentration‐Dependent Antioxidant Property

**FIGURE 1:**
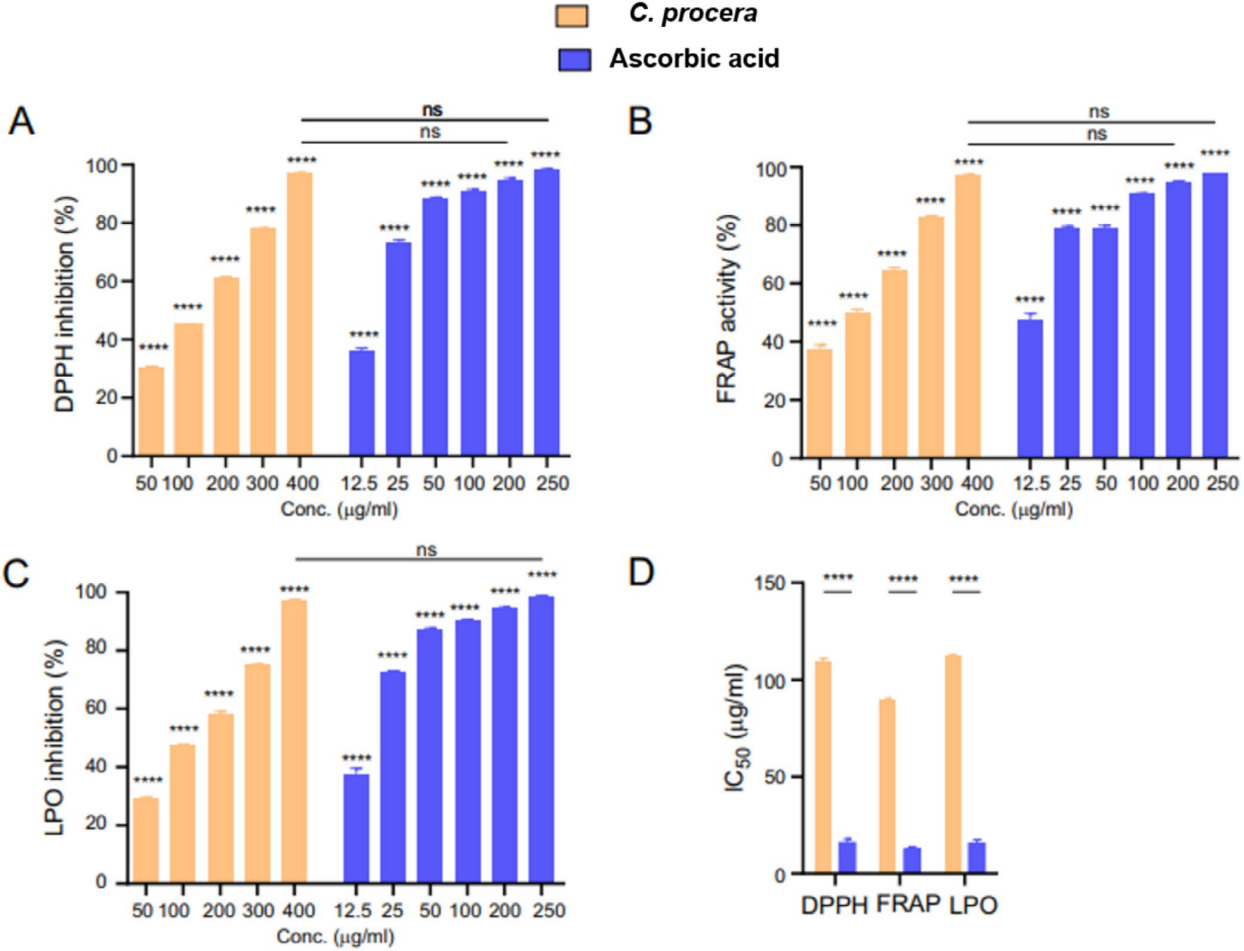
In vitro antioxidant effects of (A) 2,2‐diphenyl‐1‐picrylhydrazyl (DPPH) radicals scavenging activity of CMECP (orange) and ascorbic acid (blue) (B) Ferric‐reducing antioxidant power (FRAP) of CMECP (orange) and ascorbic acid (blue) (C) Inhibition of lipid peroxidation (LPO) of CMECP (orange) and ascorbic acid (blue) and (D) IC_50_ of CMECP and ascorbic acid. Values are presented as mean ± standard error of mean of replicate determinations. Statistical annotations: ****p < 0.0001 indicates a significant difference versus the assay blank (zero‐concentration vehicle control) for each tested concentration of CMECP and ascorbic acid; ns indicates no significant difference between CMECP at 400 μg/mL and ascorbic acid at the matched concentrations. Data are mean ± SEM of three independent determinations.

The in vitro antioxidant effects of *C. procera* extract are presented in Figure 1. Findings from this study showed that *C. procera* extract significantly (*p* < 0.05) scavenged DPPH in a concentration‐dependent manner (Figure 1A). Similarly, the extract demonstrated a significant increase in its potential to reduce ferric chloride (Figure 1B). To evaluate lipid peroxidation, the level of TRARS was measured with *C. procera* significantly inhibiting lipid peroxidation (Figure 1C). In addition, ascorbic acid, a standard antioxidant, was profiled alongside *C. procera* extract. The 50% inhibitory concentration (IC_50_) values of *C. procera* for DPPH, FRAP, and LPO were 109.6, 89.29, and 111.8 μg/mL, respectively, while the ascorbic acid had IC_50_ values of 16.08, 12.55, and 15.80 μg/mL, respectively (Figure 1D).

### 3.2 *Calotropis procera* Extract Exhibited a Concentration‐Dependent Anti‐Inflammatory Activity

**FIGURE 2:**
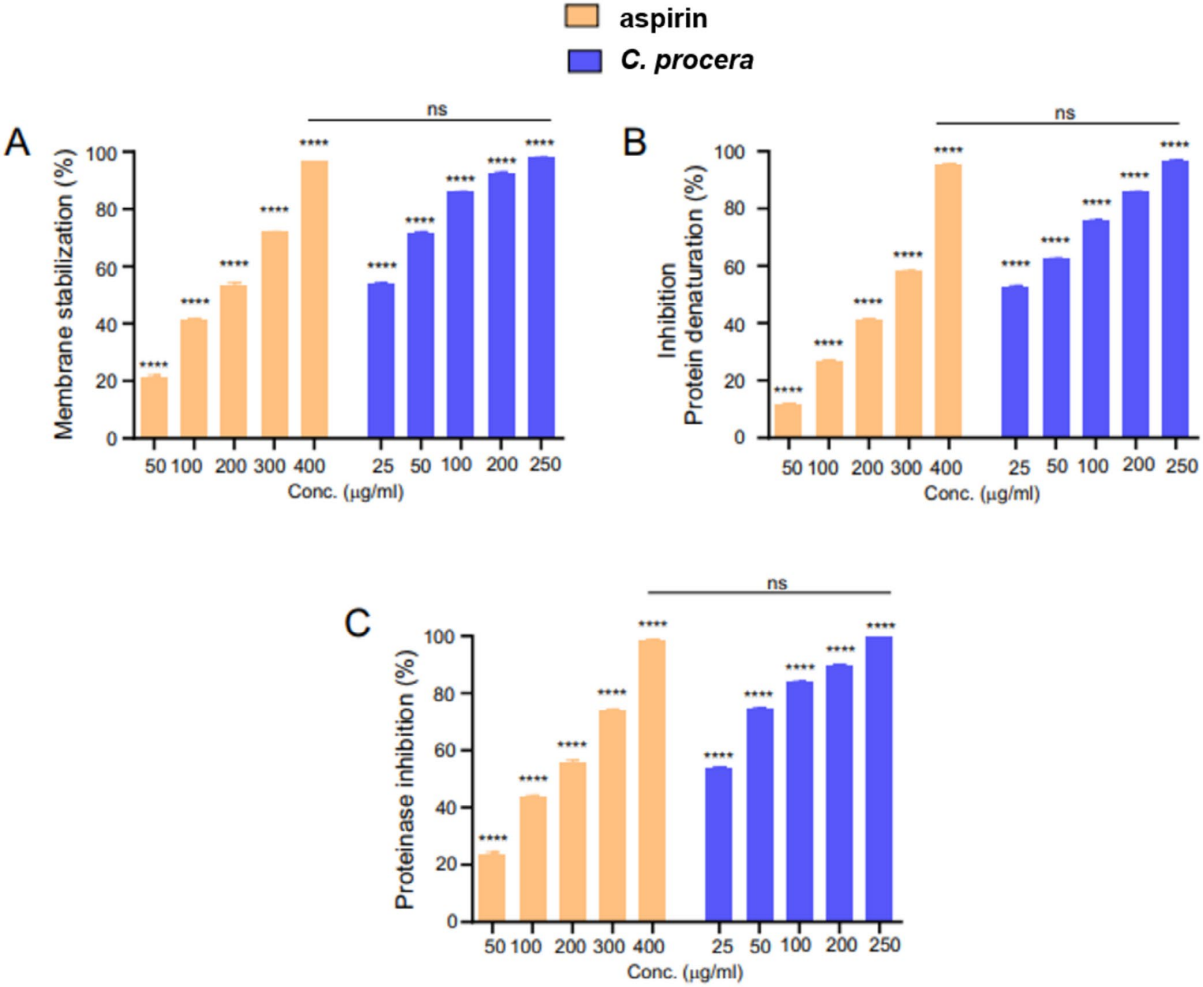
In vitro percentage of CMECP (A) membrane stabilization (blue) and aspirin, (B) protein denaturation of CMECP (blue) and aspirin (orange), and (C) proteinase inhibition of CMECP (blue) and aspirin (orange) by *C. procera* extract. Values are presented as mean ± standard error of mean of replicates determination. Statistical annotations: ****p < 0.0001 indicates a significant difference versus the assay blank (no‐enzyme/no‐substrate control) for each tested concentration of CMECP and ascorbic acid; ns indicates no significant difference between CMECP at 400 μg/mL and ascorbic acid at the matched concentrations. Data are mean ± SEM of three independent determinations.

The in vitro anti‐inflammatory effect of *C. procera* extract was examined using its percentage membrane stabilization, protein denaturation, and proteinase inhibition (Figure 2). *C. procera* significantly (*p* < 0.05) increased the stability of human red blood cell membrane (Figure 2A), inhibited protein denaturation (Figure 2B), and proteinase activity (Figure 2C) in a concentration‐dependent manner and compared favorably with the standard (aspirin). The IC_50_ values obtained were 201.6, 126.4, and 137.6 μg/mL, respectively, while aspirin had lower IC_50_ values of 26.42, 22.12, and 22.81 μg/mL, respectively.

### 3.3 *Calotropis procera* Extract Displayed Hypoglycemic Effect in a Concentration‐Dependent Manner

**FIGURE 3:**
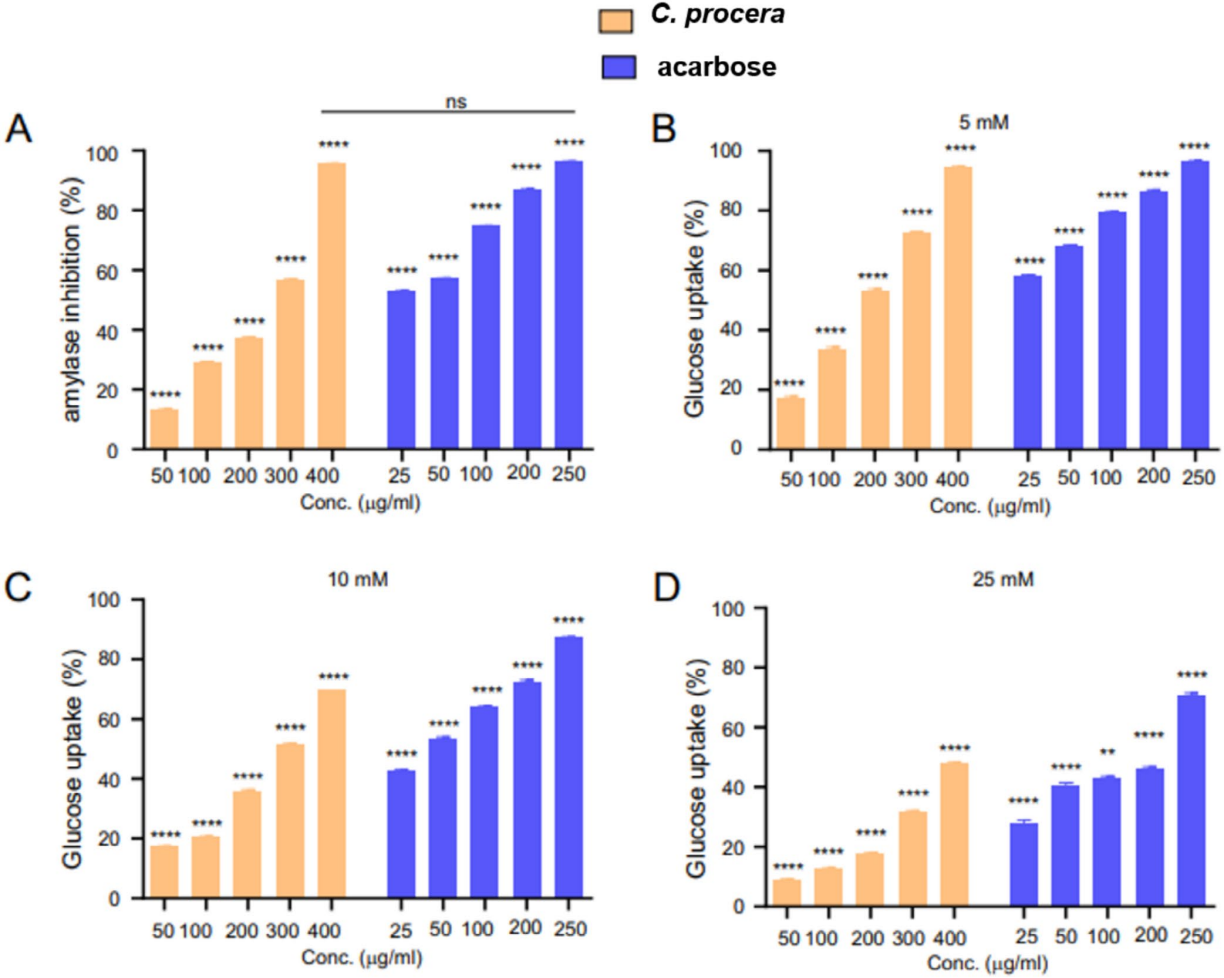
In vitro hypoglycemic effect of *C. procera* extract (A) Percentage inhibition of *α*‐amylase and glucose uptake at (B) 5, (C) 10, and (D) 25 mM concentrations of yeast cells. Values are presented as mean ± standard error of mean of replicate determinations. Statistical annotations: ****p < 0.0001 versus the assay blank for each tested concentration of CMECP and acarbose. For Panel A the blank was the α‐amylase reaction without inhibitor (full uninhibited enzyme activity); for Panels B–D the blank was the yeast‐glucose mixture incubated without extract or acarbose at the corresponding glucose concentration (5, 10 and 25 mM). ns indicates no significant difference between CMECP at 400 μg/mL and acarbose at the matched concentrations. Data are mean ± SEM of three independent determinations.

Figure 3 shows the in vitro hypoglycemic effect of *C. procera* extract using *α*‐amylase and glucose uptake in yeast cell models. *C. procera* significantly inhibited the activity of *α*‐amylase in a concentration‐dependent manner at a rate not significantly (*p* < 0.05) different from the standard (Figure 3A). In addition, in the presence of *C. procera* extract, glucose uptake by yeast cells was significantly increased at the various yeast concentrations with increasing extract concentration (Figure 3B–D). However, the percentage glucose uptake by the cells decreases as the yeast cell concentration increases, with the standard (acarbose) demonstrating more glucose uptake as the concentration increases relative to the extract.

### 3.4 *Calotropis procera* Extract Demonstrated a Hypoglycemic Effect and Improved Insulin Secretion in STZ‐Induced Diabetic Rats

**FIGURE 4:**
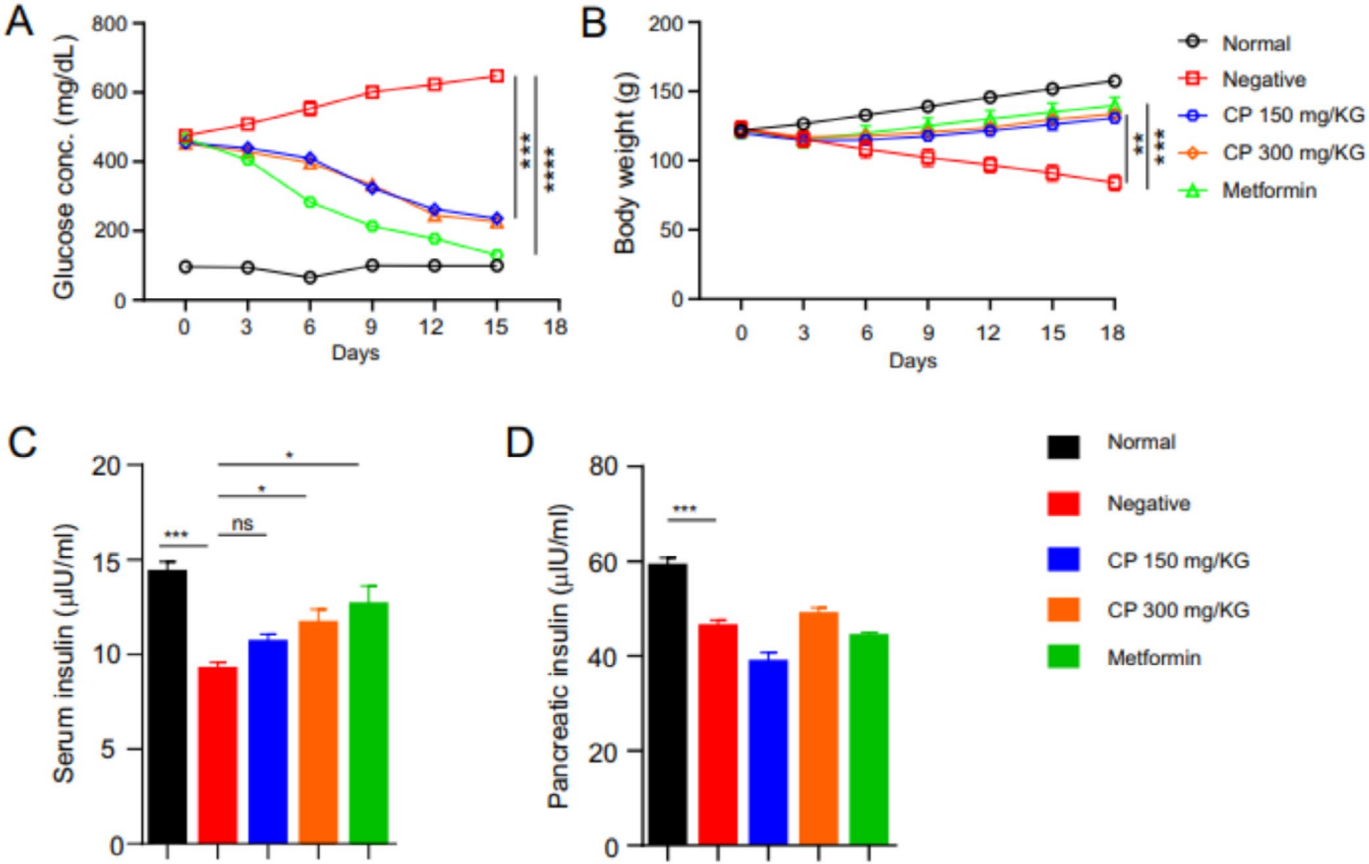
Effects of *C. procera* extract on (A) fasting blood glucose, (B) body weight, (C) serum insulin level, and (D) pancreatic insulin levels in STZ‐induced diabetic rats. Values are presented as mean ± standard error of mean of replicate determinations. *p < 0.05, **p < 0.01, ***p < 0.001, ****p < 0.0001. ns, not significant.

The hypoglycemic effect of *C. procera* extract in STZ‐induced diabetic rat model is displayed in Figure 4. Exposure of rats to STZ significantly (*p* < 0.05) increased fasting glucose level on day 0 (Figure 4A). Treatment of the STZ‐induced diabetic rats with 150 and 300 mg/Kg of *C. procera* extract and metformin progressively and significantly reduced the FBG levels over a period of 15 days (Figure 4A). While metformin showed an 80% reduction in fasting blood glucose, 300 mg/Kg of *C. procera* extract presented a 65% reduction compared to the untreated group after 15 days of treatment. Similarly, the untreated STZ‐induced diabetic rats presented a significantly (*p* < 0.05) decrease in body weight to about 38% relative to the 300 mg/kg BW treated group at the end of the treatment period. Treatment with *C. procera* extract and metformin improved the body weights of the rats (Figure 4B). While serum (Figure 4C) and pancreatic (Figure 4D) insulin levels significantly (*p* < 0.05) decreased in untreated diabetic rats compared to normal control rats, administration of *C. procera* extract and metformin significantly increased the serum insulin levels with the 300 mg/kg extract treated group showing a marginal increase in pancreas insulin relative to the untreated groups; the other treated groups presented no significant difference.

### 3.5 *Calotropis procera* Demonstrated Antioxidant Activities in STZ‐Induced Diabetic Rats

**FIGURE 5:**
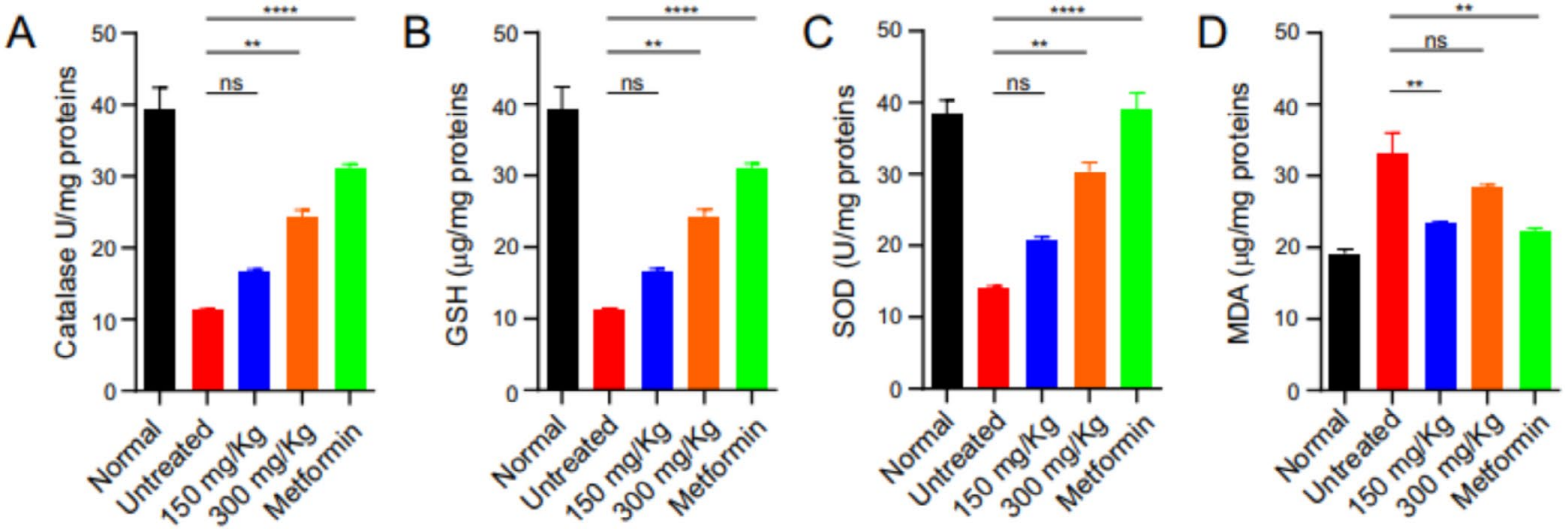
Effect of *C. procera* on (A) catalase (CAT) (B) glutathione (GSH) (C) superoxide dismutase (SOD) (D) malondialdehyde (MDA) levels in STZ‐induced diabetic rats. Values are presented as mean ± standard error of mean of replicate determinations. *p < 0.05, **p < 0.01, ***p < 0.001, ****p < 0.0001. ns, not significant.

Figure 5 shows the in vivo antioxidant activity of *C. procera* extract in STZ‐induced diabetic rats. In the diabetic untreated rats, activities of SOD, CAT, and GSH were significantly (*p* < 0.05) decreased compared to normal control (Figure 5A–C). This was also accompanied by an increase in the activities of MDA (Figure 5D). Specifically, treatment of the STZ‐induced diabetic rats with *C. procera* extract resulted in about two‐fold increases in SOD and CAT activities and GSH level at 300 mg/kg BW while also significantly (*p* < 0.05) reducing MDA levels to about 13% upon treatment with *C. procera* extract (Figure 5A–D).

### 3.6 *Calotropis procera* Reduces Hyperglycemia‐Induced Dyslipidemia in STZ‐Induced Diabetic Rats

**FIGURE 6:**
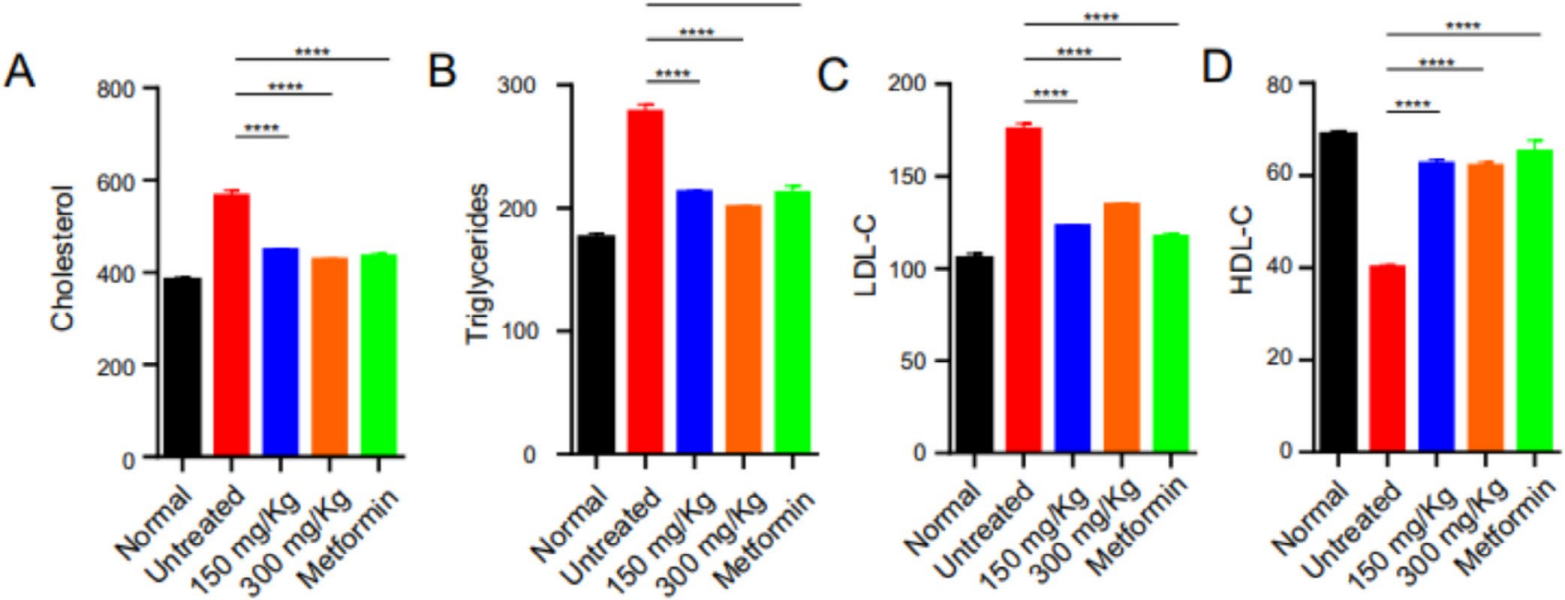
Effect of *C. procera* extract on (A) cholesterol (B) triglycerides (C) LDL‐cholesterol, and (D) HDL‐cholesterol in STZ‐induced diabetic rats. Values are presented as mean ± standard error of mean of replicate determinations. *p < 0.05, **p < 0.01, ***p < 0.001, ****p < 0.0001. ns, not significant.

The hypolipidemic effects of *C. procera* extract in STZ‐induced diabetic rats are presented in Figure 6. Upon diabetic induction, significant (*p* < 0.05) elevations in serum cholesterol (Figure 6A), triglycerides (Figure 6B), and LDL‐C (Figure 6C) levels with reduced HDL‐C level were observed (Figure 6D). However, treatment with *C. procera* extract significantly (*p* < 0.05) reversed the elevated serum level of cholesterol, triglycerides, and LDL‐C at varying percentages and significantly (*p* < 0.05) increased HDL to about 33.33% in the extract treated group; a level comparable to the normal and/or standard groups (Figure 6A–D).

### 3.7 *Calotropis procera* Reduced Neuronal Inflammatory Activities in the Brain of STZ‐Induced Diabetic Rats

**FIGURE 7:**
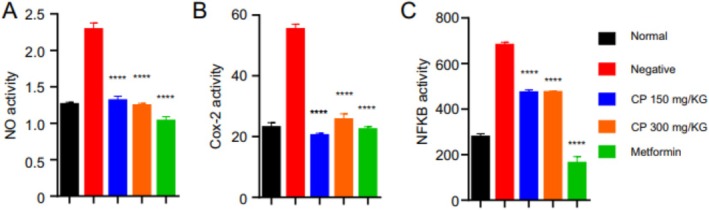
Attenuative effect of *C. procera* extract on (A) nitric oxide (NO), (B) Cox‐2, and (C) NFkB levels in the brain of STZ‐induced diabetic rats. Values are presented as mean ± standard error of mean of replicate determinations. *p < 0.05, **p < 0.01, ***p < 0.001, ****p < 0.0001 vs Negative control.

**FIGURE 8:**
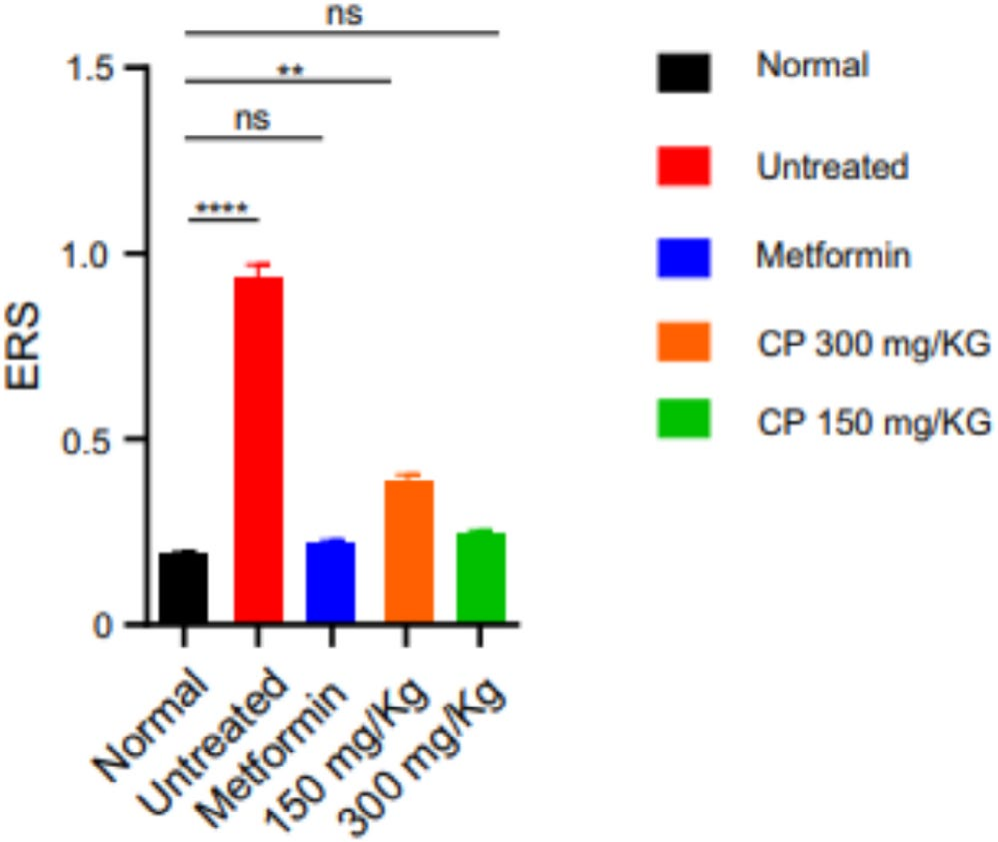
Percentage ESR of *C. procera* extract in xylene‐induced edema in the STZ‐induced diabetic rats. Values are presented as mean ± standard error of mean of replicates determination. Values in each bar with different superscripts significantly (*p* < 0.05) differ.

The attenuative effects of *C. procera* extract on STZ‐induced neuronal inflammation in the brain of diabetic rats are presented in Figure 7. STZ‐induction significantly resulted in increased expressions of Cox‐2, NF‐kB, and NO compared to the normal control (Figure 7A–C). However, treatment with *C. procera* extract significantly (*p* < 0.05) decreases the expression of the NO (Figure 7A) and Cox‐2 (Figure 7B) by about two‐folds, to levels comparable to the normal and standard groups except in NFkB levels (Figure 7C), where the levels were significantly (*p* < 0.05) higher than the control groups. Similarly, an assessment of *C. procera* extract pretreatment on xylene‐induced ear swelling in rats showed a significant (*p* < 0.05) decrease in ESR percentage compared to the untreated group (Figure 8).

### 3.8 *Calotropis procera* Effect on Cholinesterase Activities and Neurotransmitter Levels in the Brain of STZ‐Induced Diabetic Rats

**FIGURE 9:**
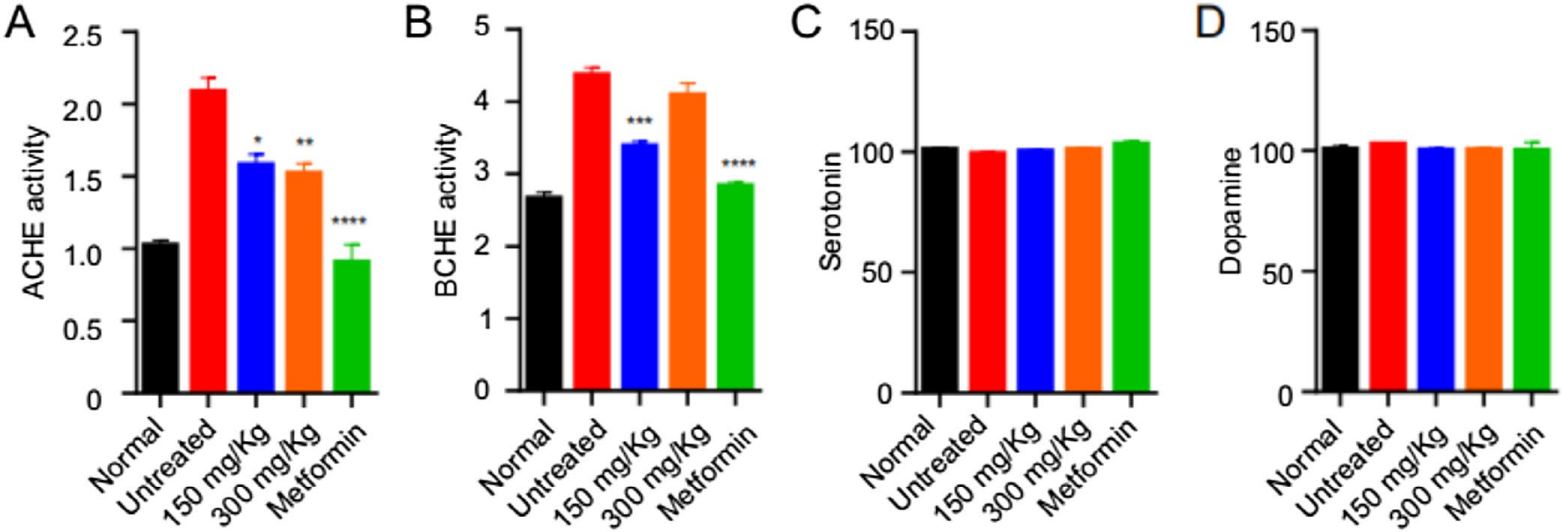
Effect of *C. procera* on (A) ACHE (B) BCHE (C) serotonin (D) dopamine levels in the brain of STZ‐induced diabetic rats. Values are presented as mean ± standard error of mean of replicates determination. *p < 0.05, **p < 0.01, ***p < 0.001, ****p < 0.0001 vs Negative control.

Figure 9 shows the modulatory effect of *C. procera* extract in the brain of STZ‐induced diabetic rats. Following diabetic induction, the concentrations of ACHE and BCHE were elevated in the untreated diabetic rats whereas a significant (*p* < 0.05) decrease in the activities of the treatment groups was observed. The decrease in BCHE activity was less prominent in the 300 mg/kg treated group (Figure 9A,B). Notably, neither the extract nor the standard drug influenced the brain's serotonin and dopamine levels (Figure 9C,D).

### 3.9 *Calotropis procera* Reversed Serum Biochemical Alterations in STZ‐Induced Diabetic Rats

Untreated diabetic rats showed significant elevations in TB, AST, ALT, ALP, urea, creatinine, uric acid and CO_3_, with reductions in total protein, albumin and potassium, relative to the normal control (Table 1). Treatment with CMECP (150 and 300 mg/kg b.wt.) and metformin (100 mg/kg b.wt.) attenuated these derangements. CMECP 300 mg/kg normalised TB and matched metformin on ALT, total protein, potassium and CO_3_ (*p* ≥ 0.05 versus normal). Both CMECP doses significantly lowered AST, ALP, urea, creatinine and uric acid versus the untreated group (*p* < 0.05 to < 0.001), though AST, ALP and albumin remained partially distinct from normal, indicating incomplete reversal at the doses tested.

**TABLE 1:** Effect of *Calotropis procera* methanolic extract on serum biochemical parameters in streptozotocin (STZ)‐induced diabetic rats.

Parameter	Normal control	Diabetic untreated	Metformin (100 mg/kg)	CMECP 150 mg/kg	CMECP 300 mg/kg	ANOVA *p*
TB	1.20 ± 0.09***	2.25 ± 0.15^###^	1.44 ± 0.06***	1.60 ± 0.18***^#^	1.19 ± 0.08***	<0.0001
DB	1.61 ± 0.19	1.22 ± 0.45	1.96 ± 0.46	1.01 ± 0.12	0.68 ± 0.09^#^	0.0036
AST	26.40 ± 2.43***	63.99 ± 4.10^###^	22.20 ± 1.98***	51.41 ± 2.14**^###^	58.91 ± 5.19^###^	<0.0001
ALT	19.05 ± 2.12*	31.11 ± 1.51^#^	15.36 ± 3.80**	25.27 ± 1.73	27.45 ± 6.09	0.0016
ALP	67.90 ± 2.50***	276.37 ± 11.86^###^	181.38 ± 1.86***^###^	165.80 ± 8.16***^###^	205.36 ± 1.78***^###^	<0.0001
TP	10.19 ± 1.06*	7.35 ± 1.57^#^	10.71 ± 0.85*	9.26 ± 0.94	10.07 ± 0.14	0.0179
ALB	6.68 ± 0.62***	3.15 ± 0.12^###^	5.27 ± 0.86**^#^	4.00 ± 0.16^###^	3.97 ± 0.13^###^	<0.0001
Na	194.70 ± 8.06	192.96 ± 9.20	199.36 ± 2.11	217.06 ± 6.14**^#^	184.71 ± 4.43	0.0015
K	14.38 ± 1.59**	8.05 ± 1.81^##^	13.83 ± 1.48*	11.27 ± 2.31	11.98 ± 0.74	0.0066
CO3	17.72 ± 1.17*	23.96 ± 2.85^#^	22.25 ± 1.59	20.86 ± 1.85	16.97 ± 1.20**	0.0040
Urea	21.02 ± 1.54***	42.86 ± 8.25^###^	28.72 ± 1.58*	27.61 ± 0.68**	33.63 ± 2.61^#^	0.0007
Creatinine	4.59 ± 0.57***	10.18 ± 0.46^###^	5.23 ± 0.34***	5.79 ± 0.33***^#^	5.71 ± 0.30***^#^	<0.0001
Uric acid	27.61 ± 1.07***	42.28 ± 4.29^###^	32.54 ± 0.64**	35.66 ± 2.95^#^	31.55 ± 1.40**	0.0003

*Note:* Values are presented as mean ± standard deviation (SD) of *n* = 3 rats per group. Data were analysed by one‐way ANOVA followed by Tukey's honestly significant difference (HSD) post‐hoc test using GraphPad Prism v8.0. The ANOVA *p*‐value reported in the right‐hand column refers to the global between‐group test. Pairwise significance is indicated as: * *p* < 0.05, ** *p* < 0.01, *** *p* < 0.001 versus the diabetic untreated group; and ^**#**^ *p* < 0.05, ^**##**^ *p* < 0.01, ^**###**^ *p* < 0.001 versus the normal (non‐diabetic) control. Absence of a symbol denotes *p* ≥ 0.05. CMECP, crude methanolic extract of *Calotropis procera*. Units: TB and DB, mg/dL; AST, ALT and ALP, U/L; TP and ALB, g/dL; Na, K and CO_3_, mmol/L; urea, creatinine and uric acid, mg/dL.

### 3.10 *Calotropis procera* Ameliorated the Hematological Alterations in STZ‐Induced Diabetics in Rats

Untreated diabetic rats had significantly reduced haemoglobin (Hb) and lymphocyte fraction with a reciprocal rise in total white blood cells (TWBC) versus the normal control (Table 2). CMECP 150 mg/kg restored Hb and the lymphocyte fraction, while metformin produced the strongest correction of Hb, TWBC, neutrophil, lymphocyte and monocyte fractions (*p* < 0.05 to < 0.01 versus untreated). MCHC and the eosinophil fraction were unchanged across groups. Trends in PCV, MCV, MCH, RBC and platelets were observed but did not reach statistical significance at the current sample size, and should be interpreted as descriptive only.

**TABLE 2:** Effect of *Calotropis procera* methanolic extract on haematological parameters in streptozotocin (STZ)‐induced diabetic rats.

Parameter	Normal control	Diabetic untreated	Metformin (100 mg/kg)	CMECP 150 mg/kg	CMECP 300 mg/kg	ANOVA *p*
Hb	16.33 ± 0.67**	9.33 ± 1.43^##^	15.27 ± 1.75**	13.20 ± 1.20	12.40 ± 1.95	0.0014
PCV	40.17 ± 0.96	30.57 ± 2.15	40.23 ± 3.82	39.00 ± 6.56	40.50 ± 6.54	0.1043
MCV	78.60 ± 4.81	90.13 ± 2.61	77.00 ± 2.98	79.00 ± 10.54	74.00 ± 9.00	0.1150
MCH	25.90 ± 1.28	21.83 ± 1.53	24.00 ± 4.88	25.00 ± 5.00	27.50 ± 4.50	0.4880
MCHC	29.20 ± 1.39	22.93 ± 1.31	26.93 ± 0.96	23.17 ± 4.02	23.00 ± 6.56	0.1797
RBC	5.91 ± 0.85	3.59 ± 0.54	4.63 ± 1.47	3.90 ± 1.25	4.80 ± 0.78	0.1316
PLC	173.00 ± 10.54	185.67 ± 8.33	173.00 ± 6.56	174.00 ± 9.54	168.50 ± 10.83	0.2929
TWBC	97.00 ± 2.38*	115.37 ± 5.49^#^	95.67 ± 6.74*	113.50 ± 4.27^#^	107.00 ± 7.55	0.0037
N	48.73 ± 2.15	59.00 ± 9.72	40.00 ± 4.16*	50.00 ± 4.58	47.00 ± 5.29	0.0295
L	50.80 ± 1.08*	32.03 ± 3.27^#^	53.33 ± 5.50**	44.50 ± 9.10	43.50 ± 4.85	0.0062
M	4.80 ± 0.80	2.63 ± 1.10	5.47 ± 1.10*	4.50 ± 1.00	5.00 ± 0.50	0.0313
E	4.60 ± 1.14	3.67 ± 1.36	4.53 ± 1.55	6.50 ± 0.50	4.00 ± 1.06	0.0990

*Note:* Values are presented as mean ± standard deviation (SD) of *n* = 3 rats per group. Data were analysed by one‐way ANOVA followed by Tukey's honestly significant difference (HSD) post‐hoc test using GraphPad Prism v8.0. The ANOVA *p*‐value reported in the right‐hand column refers to the global between‐group test. Pairwise significance is indicated as: *p < 0.05, **p < 0.01, ***p < 0.001 versus the diabetic untreated group; and ^#^ *p* < 0.05, ^##^ *p* < 0.01, ^###^ *p* < 0.001 versus the normal (non‐diabetic) control. Absence of a symbol denotes *p* ≥ 0.05. CMECP, crude methanolic extract of *Calotropis procera*. Units and abbreviations: Hb (haemoglobin), g/dL; PCV (packed cell volume), %; MCV (mean cell volume), fL; MCH (mean cell haemoglobin), pg; MCHC (mean cell haemoglobin concentration), g/dL; RBC (red blood cells), ×10^12^/L; PLC (platelets), ×10^9^/L; TWBC (total white blood cells), ×10^9^/L; N (neutrophils), L (lymphocytes), M (monocytes) and E (eosinophils), % of total leucocytes.

## 4 Discussion

The rising morbidity and mortality rate arising from diabetes associated complications demands the development of more effective and safe therapeutic options. Despite the efficacy of the current arsenal of synthetic antidiabetic drugs, their adverse effects necessitate alternative drug development. Natural products including plants are beneficial in this regard due to their rich natural reservoir of chemical compounds with less adverse effects (Salehi et al. 2019). Several bioactive compounds belonging to flavonoids, terpenoids, anthocyanins, saponins, glycosides, tannins, steroids, cardenolide, alkaloids, and resins present in *C. procera* have been attributed to its biological properties including antidiabetic effects (Okwudiri Ihegboro et al. 2022). Based on this, this study evaluated the effect of *C. procera*, an understudied potential antidiabetic candidate, for its role in diabetes complications.

Free radicals' formation and the accompanying oxidative stress have been extensively implicated in the progression of T2DM. Therefore, potent antioxidants capable of scavenging free radicals are essential in curbing diabetic progression. Thus, the study assessed the antioxidant potentials of *C. procera* extract in vitro by measuring its ability to scavenge DPPH, reduce insoluble ferric (Fe^3+^) to soluble ferrous (Fe^2+^) and LPO. The observed free radicals scavenging capacity of the extract suggests its antioxidant efficacy. This is consistent with the study of (Patel et al. 2014) who attributed the phenolics and tannins content of *C. procera* to its antioxidant activity (Banerjee et al. 2016). Furthermore, oxidative stress plays a key role in the progression and development of diabetic complications such as diabetic retinopathy, hypertension, cardiomyopathy, nephropathy, and neuropathies (Dattatreya and Sarangi 2015). To investigate the interplay between antidiabetic drug and ability to scavenge free radicals both in vitro and in vivo, metformin, a standard antidiabetic drug, was profiled alongside *C. procera* extract. Although metformin had a significantly reduced IC_50_ which might be due to its existence as a pure compound rather than a mixture of compounds in the extract. Administration of *C. procera* extract in STZ‐induced diabetic rats resulted in improved antioxidant activities compared to the untreated groups.

Given the correlation between diabetes, oxidative stress, and inflammation, the study investigated the anti‐inflammatory activity of *C. procera* in addition to antioxidant properties (Rahmatullah et al. 2013; Shewamene et al. 2015; Salehi et al. 2019). Interestingly, the extract demonstrated anti‐inflammatory effects comparable to those of the standard drug. Although standard drug exhibited significantly lower IC_50_ compared to the extract, the anti‐inflammatory effects displayed by the extract suggest their potential to mitigate membrane destabilization, protein denaturation and enhance proteinase inhibition. Consistent with previous reports that hyperglycemic‐triggered inflammation is mediated by the excessive release of inflammatory mediators in the brain (Rübsam et al. 2018; Sobeh et al. 2019). Measurement of the levels of expression of inflammatory mediators in the brains of diabetic rats indicate that *C. procera* extract possibly exhibits anti‐inflammatory effects via inhibition of inflammatory mediators such as Cox‐2, NF‐kB and NO. This agrees with the study of (Karale et al. 2021) where they showed that *C. procera* exhibits in vitro and in vivo anti‐inflammatory activities with the methanolic extract presenting the highest activity among the other tested solvent extracts. Similarly, pretreatment of the diabetic rats with *C. procera* extract significantly prevented xylene‐induced ear swelling compared to the untreated control. All together, these results indicate that *C. procera* possess anti‐inflammatory properties capable of protecting tissues against diabetic‐induced inflammation.

Potential antidiabetic drug candidates should be able to regulate blood glucose levels by inhibiting carbohydrate‐metabolizing enzymes such as amylase, improving cellular glucose uptake and utilization, and enhancing insulin sensitivity. Therefore, the potentials of *C. procera* in inhibiting amylase and enhancing glucose absorption in the mammalian intestinal lumen were investigated using an in vitro yeast cell model. The extract significantly inhibited α‐amylase activity and increased glucose uptake in yeast cells, indicating its potential hypoglycemic effect. This was further supported by in vivo analysis, where diabetic rats treated with the extract showed reduced fasting blood sugar levels and improved body weights, which is consistent with the study of (Neto et al. 2020). This observation underscores the extract's ability to reduce postprandial blood glucose levels and ability to mobilize the transport of glucose to the cells for energy generation via glycolysis or storage of excess glucose via glycogenesis and lipogenesis, hence the observed increment in body weight. Additionally, the observed increase in serum insulin levels relative to pancreatic insulin levels could indicate improved insulin production and activity in the pancreas and serum, respectively.

Persistent hyperglycemia results in alterations in liver and kidney functions which may progress to organ failure (Jeong et al. 2020). Fortunately, administration of *C. procera* extract to STZ‐induced diabetic rats normalised serum total bilirubin and significantly lowered ALP, urea, creatinine and uric acid, with AST also reduced at the 150 mg/kg dose. Total protein and albumin were partially restored, although full normalisation of these synthetic liver markers was achieved only with metformin, suggesting that CMECP at the doses tested attenuates rather than abolishes the hepatic synthetic deficit caused by STZ. Similarly, the alterations in kidney biomarkers [creatinine, urea, and electrolytes] resulting from STZ induction were reversed upon *C. procera* treatment to levels comparable to the normal and/or standard suggesting possible reduction in excessive urination and thirst associated with T2DM (Eze et al. 2019). Dysmetabolism of lipid in T2DM usually presents high serum levels of cholesterol, triglycerides (TGs), low‐density lipoprotein‐cholesterol (LDL‐C), and low levels of high‐density lipoprotein‐cholesterol (HDL‐C) (Al‐Trad et al. 2019; Lehmann‐Werman et al. 2018). Here, treatment with 150 and 300 mg/kg of *C. procera* extract reversed the alterations in lipid parameters upon STZ administration depicting a robust efficacy of *C. procera* in attenuating diabetic dyslipidemia. Hematological parameters are pointers to pathological conditions such as anemia, erythropoietin production, glycosylation reactions, immune responses, or can be an indicator for diabetic foot ulcer in diabetic patients (Akanji et al. 2023; Sen et al. 2005). The ability of *C. procera* to reverse the alterations caused by STZ potentiates its ability to improve diabetic‐induced hematological changes. Collectively, *C. procera* extract effectively reversed the biochemical dysfunctions that accompany STZ‐induced diabetics.

Elevated concentrations of acetylcholinesterase (ACHE) and butyrylcholinesterase (BCHE) in tissue and plasma are usually observed in T2DM and Alzheimer's disease (Rao et al. 2007; Hogan et al. 2023; Secnik et al. 2020). These elevations result in a hydrolysis reaction that reduces acetylcholine and butyrylcholine levels, leading to low‐grade systemic inflammatory responses normally presented in T2DM and Alzheimer's patients (Mushtaq et al. 2014). Treatment of diabetic rats with *C. procera* extract reduced the activity of ACHE and BCHE, possibly preventing diabetic‐induced neurological diseases such as Alzheimer's (Mushtaq et al. 2014; Vaknine and Soreq 2020). Serotonin improves β‐cells' response to glucose (Pérez‐Taboada et al. 2020), however, hyperglycemia portends a decrease in neurotransmitter release, thus β‐cells' response to glucose (Zhou et al. 2016). The non‐significant effect of the extract at the tested doses on serotonin and dopamine levels suggests its non‐modulatory effects on β‐cells. Overall, this preliminary study supports the potentials of *C. procera* against oxidative stress, inflammation, diabetes, and diabetes‐related complications.

## 5 Conclusions

The study findings suggest that *C. procera* has the potential to improve hyperglycemia and its related complications both in vitro and in vivo. Given the connection between oxidative stress, inflammation, and diabetic complications, substances with strong antioxidant properties could play a crucial role in preventing diabetic complications. *C. procera* , known for its hypoglycemic and antioxidant properties, showed promise in reducing diabetic‐induced complications. The plant's rich bioactive compounds, particularly flavonoids, are likely to contribute to its overall biological effects. These results support the idea that *C. procera* could be a valuable therapeutic agent for diabetes and its complications. Although the present study reports antioxidants, anti‐inflammatory, and anti‐diabetic potential of *C. procera* , studies tailored towards the identification of the specific compounds contributing to the observed biological activities as well as the identification of hit compounds that could be studied further as potential antidiabetic drug candidates are in the pipeline.

## Author Contributions

**Yu‐Cheng Kuo:** conceptualization, methodology, formal analysis, software, investigation, writing – original draft preparation, writing – reviewing and editing. **Musa I. Ajibola:** conceptualization, methodology, formal analysis, software, investigation, validation, writing – original draft preparation, writing – reviewing and editing. **Bashir Lawal:** conceptualization, methodology, software, validation, writing – original draft preparation, writing – reviewing and editing. **Halimat Y. Lukman:** conceptualization, methodology, formal analysis, writing – original draft preparation, writing – reviewing and editing. **Lung‐Ching Chen:** data curation, investigation, software, validation, writing – reviewing and editing. **Sheng‐Liang Huang:** conceptualization, formal analysis, software, supervision, writing – reviewing and editing. **Olawale David Ajayi:** conceptualization, formal analysis, software, supervision, writing – reviewing and editing. **Sunday Amos Onikanni:** data curation, investigation, software, validation, supervision, funding, writing – reviewing and editing. **Saheed Sabiu:** methodology, formal analysis, writing – reviewing and editing. **Leandro Miranda‐Alves:** conceptualization, methodology, formal analysis, software, funding. **Hsu‐Shan Huang:** conceptualization, methodology, formal analysis, software, funding, writing – original draft preparation. All authors have read and agreed to the published version of the manuscript.

## Funding

This research was supported by Taipei Medical University (112TMU‐WFH‐06, DP2‐TMU‐113‐O‐06, SKH‐TMU‐113‐02, SKH‐TMU‐114‐04, 114_T&N_07 and A‐112‐071). The Article Processing Charge for the publication of this research was funded partially by the Coordenacao de Aperfeicoamento de Pessoal de Nivel Superior—Brasil (CAPES) (ROR identifier: 00x0ma614).

## Conflicts of Interest

The authors declare no conflicts of interest.

## Data Availability

The data that support the findings of this study are available on request from the corresponding author. The data are not publicly available due to privacy or ethical restrictions.

## References

[fsn371364-bib-0001] Abdulrasheed‐Adeleke, T. , B. Lawal , E. I. Agwupuye , et al. 2023. “Apigetrin‐Enriched Pulmeria Alba Extract Prevents Assault of STZ on Pancreatic β‐Cells and Neuronal Oxidative Stress With Concomitant Attenuation of Tissue Damage and Suppression of Inflammation in the Brain of Diabetic Rats.” Biomedicine & Pharmacotherapy 162: 114582. 10.1016/j.biopha.2023.114582. 36989727

[fsn371364-bib-0002] Adewuyi, H. A. , A. Y. Kabiru , H. L. Muhammad , et al. 2024. “Pre‐Clinical Protective Potentials of *Carica papaya* Constituents in Experimentally Induced Anemia.” American Journal of Translational Research 16, no. 7: 3259. 10.62347/ZQDC9694. 39114700 PMC11301493

[fsn371364-bib-0003] Ahmad Nejhad, A. , B. Alizadeh Behbahani , M. Hojjati , A. Vasiee , and M. A. Mehrnia . 2023. “Identification of Phytochemical, Antioxidant, Anticancer and Antimicrobial Potential of *Calotropis procera* Leaf Aqueous Extract.” Scientific Reports 13, no. 1: 14716. 10.1038/s41598-023-42086-1. 37679486 PMC10485245

[fsn371364-bib-0004] Akanji, O. C. , B. O. Gabriel , E. O. Oshomoh , and O. S. Asuelimen . 2023. “Antidiabetic Property of *Chrysophyllum albidum* Extract in Streptozotozin‐Induced Diabetic Rats.” Journal of Phytopharmacology 9, no. 4: 230–235. 10.31254/phyto.2020.9403.

[fsn371364-bib-0005] Albers, J. J. , G. R. Warnick , and M. C. Chenng . 1978. “Quantitation of High Density Lipoproteins.” Lipids 13, no. 12: 926–932. 10.1007/BF02533852. 220488

[fsn371364-bib-0006] Al‐Trad, B. , H. Alkhateeb , W. Alsmadi , and M. Al‐Zoubi . 2019. “Eugenol Ameliorates Insulin Resistance, Oxidative Stress and Inflammation in High Fat‐Diet/Streptozotocin‐Induced Diabetic Rat.” Life Sciences 216: 183–188. 10.1007/s11033-022-08108-3. 30448265

[fsn371364-bib-0007] American Diabetes Association Professional Practice Committee, & American Diabetes Association Professional Practice Committee . 2022. “Classification and Diagnosis of Diabetes: Standards of Medical Care in Diabetes—2022.” Diabetes Care 45, no. 1: S17–S38. 10.2337/cd17-0119. 34964875

[fsn371364-bib-0008] Arumugam, G. , P. Manjula , and N. Paari . 2013. “A Review: Anti Diabetic Medicinal Plants Used for Diabetes Mellitus.” Journal of Acute Disease 2, no. 3: 196–200. 10.1016/S2221-6189(13)60126-2.

[fsn371364-bib-0009] Banerjee, S. H. A. R. M. I. S. T. H. A. , S. H. U. C. H. I. Kaushik , and R. S. Tomar . 2016. “Effect of Different Solvents on Antioxidant Activity of Leaf Extracts of Calotropis Procera and *Azadirachta indica* .” Asian Journal of Pharmaceutical and Clinical Research 10: 1. 10.22159/ajpcr.2017.v10i1.15145.

[fsn371364-bib-0010] Cirillo, V. P. 1962. “Mechanism of Glucose Transport Across the Yeast Cell Membrane.” Journal of Bacteriology 84, no. 3: 485–491. 10.1128/jb.84.3.485-491.1962. 14021412 PMC277903

[fsn371364-bib-0011] Dacie, J. , and S. Lewis . 1991. Practical Textbook of Haematology 7th Edition Edinburgh. Vol. 7, 54–79. Church Livingstone.

[fsn371364-bib-0012] Dao, T. N. P. , S. A. Onikanni , A. O. Fadaka , et al. 2025. “Investigating the Potential of the Chinese Herbal Remedy SS‐1 for the Treatment of Sjögren's Syndrome via the Interleukin‐17 Signaling Axis.” Journal of Traditional and Complementary Medicine. 10.1016/j.jtcme.2025.04.001. PMC13083303 42006484

[fsn371364-bib-0013] Dattatreya, A. , and T. K. Sarangi . 2015. “A Review on Diabetes Mellitus: Complications, Management and Treatment Modalities.” Research & Reviews. Journal of Medical and Health Sciences 4, no. 3: 10. 10.36106/ijar/8000731.

[fsn371364-bib-0014] Delanghe, J. R. , and M. M. Speeckaert . 2011. “Creatinine Determination According to Jaffe—What Does It Stand for?” Clinical Kidney Journal 4, no. 2: 83–86. 10.1093/ndtplus/sfq211. PMC4421578 25984118

[fsn371364-bib-0015] Doumas, B. T. , W. A. Watson , and H. G. Biggs . 1971. “Albumin Standards and the Measurement of Serum Albumin With Bromcresol Green.” Clinica Chimica Acta 31, no. 1: 87–96. 10.1016/0009-8981(71)90365-2. 5544065

[fsn371364-bib-0016] Ellman, G. L. , K. D. Courtney , V. Andres Jr. , and R. M. Featherstone . 1961. “A New and Rapid Colorimetric Determination of Acetylcholinesterase Activity.” Biochemical Pharmacology 7, no. 2: 88–95. 10.1016/0006-2952(61)90145-9. 13726518

[fsn371364-bib-0017] Eze, E. D. , A. M. Afodun , J. Kasolo , and K. I. Kasozi . 2019. “Lycopene Improves on Basic Hematological and Immunological Parameters in Diabetes Mellitus.” BMC Research Notes 12, no. 1: 805. 10.1186/s13104-019-4841-8. 31831054 PMC6909647

[fsn371364-bib-0018] Friedewald, W. T. , R. I. Levy , and D. S. Fredrickson . 1972. “Estimation of the Concentration of Low‐Density Lipoprotein Cholesterol in Plasma, Without Use of the Preparative Ultracentrifuge.” Clinical Chemistry 18, no. 6: 499–502. 10.1093/clinchem/18.6.499. 4337382

[fsn371364-bib-0019] Gl, E. 1959. “Tissue Sulfhydryl Groups.” Archives of Biochemistry and Biophysics 82: 70–77. 10.1016/0003-9861(59)90090-6. 13650640

[fsn371364-bib-0020] Gornall, A. G. , C. J. Bardawill , and M. M. David . 1949. “Determination of Serum Proteins by Means of the Biuret Reaction.” Journal of Biological Chemistry 177, no. 2: 751–766 PMID: 18110453. 18110453

[fsn371364-bib-0021] Hernández‐Montañez, Z. , M. Juárez‐Montiel , M. Velázquez‐Ávila , et al. 2012. “Production and Characterization of Extracellular α‐Amylase Produced by Wickerhamia sp. X‐Fep.” Applied Biochemistry and Biotechnology 167, no. 7: 2117–2129. 10.1007/s12010-012-9736-2. 22678824

[fsn371364-bib-0022] Hogan, I. A. , Y. C. Kuo , A. N. Abubakar , et al. 2023. “Attenuation of Hyperglycemia‐Associated Dyslipidemic, Oxidative, Cognitive, and Inflammatory Crises via Modulation of Neuronal ChEs/NF‐κB/COX‐2/NOx, and Hepatorenal Functional Deficits by the *Tridax procumbens* Extract.” Biomedicine & Pharmacotherapy 158: 114114. 10.1016/j.biopha.2022.114114. 36525818

[fsn371364-bib-0023] Janssen, L. M. M. , M. Hiligsmann , A. M. J. Elissen , et al. 2020. “Burden of Disease of Type 2 Diabetes Mellitus: Cost of Illness and Quality of Life Estimated Using the Maastricht Study.” Diabetic Medicine 37, no. 10: 1759–1765. 10.1111/dme.14285. 32112462 PMC7539911

[fsn371364-bib-0024] Jeong, E. , N. Park , Y. Kim , J. Y. Jeon , W. Y. Chung , and D. Yoon . 2020. “Temporal Trajectories of Accompanying Comorbidities in Patients With Type 2 Diabetes: A Korean Nationwide Observational Study.” Scientific Reports 10, no. 1: 5535. 10.1038/s41598-020-62482-1. 32218498 PMC7099011

[fsn371364-bib-0025] Karale, P. , S. Dhawale , M. Karale , and T. Kadam . 2021. “Effect of *Calotropis procera* Leaf Extracts and Partitioned Fractions on Anti‐Inflammatory and Analgesic Activity.” International Journal of Botany 6, no. 4: 862–867.

[fsn371364-bib-0026] Karale, P. A. , and M. A. Karale . 2017. “A Review on Phytochemistry and Pharmacological Properties of Milkweed Family Herbs (Asclepiadaceae).” Asian Journal of Pharmaceutical and Clinical Research 10, no. 11: 27–34. 10.22159/ajpcr.2017.v10i11.21215.

[fsn371364-bib-0027] Lawal, B. , S. Sani , A. S. Onikanni , et al. 2022. “Preclinical Anti‐Inflammatory and Antioxidant Effects of Azanza Garckeana in STZ‐Induced Glycemic‐Impaired Rats, and Pharmacoinformatics of It Major Phytoconstituents.” Biomedicine & Pharmacotherapy 152: 113196. 10.1016/j.biopha.2022.113196. 35667233

[fsn371364-bib-0028] Lehmann‐Werman, R. , J. Magenheim , J. Moss , et al. 2018. “Monitoring Liver Damage Using Hepatocyte‐Specific Methylation Markers in Cell‐Free Circulating DNA.” JCI Insight 3, no. 12: e120687. 10.1172/jci.insight.120687. 29925683 PMC6124429

[fsn371364-bib-0029] Lukman, H. Y. , Y. Kuo , M. S. Owolabi , et al. 2024. “Evaluation of Terpenes Rich *Hura crepitans* Extract on Glucose Regulation and Diabetic Complications in STZ‐Induced Diabetic Rats.” Biomedicine & Pharmacotherapy 179: 117308. 10.1016/j.biopha.2024.117308. 39180791

[fsn371364-bib-0030] Malek, R. , S. Hannat , A. Nechadi , F. Z. Mekideche , and M. Kaabeche . 2019. “Diabetes and Ramadan: A Multicenter Study in Algerian Population.” Diabetes Research and Clinical Practice 150: 322–330. 10.1016/j.diabres.2019.02.008. 30779972

[fsn371364-bib-0031] Mezil, S. A. , and B. A. Abed . 2021. “Complication of Diabetes Mellitus.” Annals of the Romanian Society for Cell Biology 25, no. 3: 1546–1556.

[fsn371364-bib-0032] Miranda, K. M. , M. G. Espey , and D. A. Wink . 2001. “A Rapid, Simple Spectrophotometric Method for Simultaneous Detection of Nitrate and Nitrite.” Nitric Oxide 5, no. 1: 62–71. 10.1006/niox.2000.0319. 11178938

[fsn371364-bib-0033] Misra, H. P. , and I. Fridovich . 1972. “The Role of Superoxide Anion in the Autoxidation of Epinephrine and a Simple Assay for Superoxide Dismutase.” Journal of Biological Chemistry 247, no. 10: 3170–3175. 4623845

[fsn371364-bib-0034] Mizushima, Y. , and M. Kobayashi . 1968. “Interaction of Anti‐Inflammatory Drugs With Serum Proteins, Especially With Some Biologically Active Proteins.” Journal of Pharmacy and Pharmacology 20, no. 3: 169–173. 10.1111/j.2042-7158.1968.tb09718.x. 4385045

[fsn371364-bib-0035] Mushtaq, G. , N. A. H Greig , J. Khan , and M. A Kamal . 2014. “Status of Acetylcholinesterase and Butyrylcholinesterase in Alzheimer's Disease and Type 2 Diabetes Mellitus.” CNS & Neurological Disorders‐Drug Targets (Formerly Current Drug Targets‐CNS & Neurological Disorders) 13, no. 8: 1432–1439. 10.2174/1871527313666141023141545. PMC5878042 25345511

[fsn371364-bib-0036] Neto, L. S. , R. Q. Moraes‐Souza , T. S. Soares , et al. 2020. “A Treatment With a Boiled Aqueous Extract of *Hancornia speciosa* Gomes Leaves Improves the Metabolic Status of Streptozotocin‐Induced Diabetic Rats.” BMC Complementary Medicine and Therapies 20, no. 1: 114. 10.1186/s12906-020-02919-2. 32303220 PMC7164147

[fsn371364-bib-0037] Neto, M. C. , C. F. de Vasconcelos , V. N. Thijan , et al. 2013. “Evaluation of Antihyperglycaemic Activity of *Calotropis procera* Leaves Extract on Streptozotocin‐Induced Diabetes in Wistar Rats.” Revista Brasileira de Farmacognosia 23, no. 6: 913–919. 10.1590/S0102-695X2013000600008.

[fsn371364-bib-0038] Okwudiri Ihegboro, G. , T. Alowonle Owolarafe , C. James Ononamadu , H. Bello , and M. Kufre‐Akpan . 2022. “ *Calotropis Procera* Root Extract's Anti‐Diabetic and Hepatoprotective Therapeutic Activity in Alloxan‐Induced Pancreatic Toxicity in Wistar Rats.” Iran Journal of Toxicology 16, no. 4: 285–296. 10.32598/IJT.16.4.966.1.

[fsn371364-bib-0039] Oloyede, H. O. , H. Y. Lukman , and M. O. Salawu . 2020. “Protective Potentials of Ethyl Acetate‐Ethanolic Fraction of *Carica papaya* Leaves Against Acetaminophen‐Induced Liver Damage in Rats.” Notulae Scientia Biologicae 12, no. 3: 556–567. 10.15835/nsb12310629.

[fsn371364-bib-0040] Ong, K. L. , L. K. Stafford , S. A. McLaughlin , et al. 2023. “Global, Regional, and National Burden of Diabetes From 1990 to 2021, With Projections of Prevalence to 2050: A Systematic Analysis for the Global Burden of Disease Study 2021.” Lancet 402, no. 10397: 203–234. 10.1016/s0140-6736(23)01301-6. 37356446 PMC10364581

[fsn371364-bib-0041] Onikanni, A. S. , B. Lawal , A. O. Olusola , et al. 2021. “ *Sterculia tragacantha* Lindl Leaf Extract Ameliorates STZ‐Induced Diabetes, Oxidative Stress, Inflammation and Neuronal Impairment.” Journal of Inflammation Research 14: 6749–6764. 10.2147/JIR.S319673. 34916823 PMC8668250

[fsn371364-bib-0042] Onikanni, A. S. , B. Lawal , B. E. Oyinloye , et al. 2022. “Therapeutic Efficacy of Clompanus Pubescens Leaves Fractions via Downregulation of Neuronal Cholinesterases/Na+‐K+ ATPase/IL‐1 β, and Improving the Neurocognitive and Antioxidants Status of Streptozotocin‐Induced Diabetic Rats.” Biomedicine & Pharmacotherapy 148: 112730. 10.1016/j.biopha.2022.112730. 35183996

[fsn371364-bib-0043] Onikanni, S. A. , B. Lawal , V. Munyembaraga , et al. 2023. “Profiling the Antidiabetic Potential of Compounds Identified From Fractionated Extracts of Entada Africana Toward Glucokinase Stimulation: Computational Insight.” Molecules 28, no. 15: 5752. 10.3390/molecules28155752. 37570723 PMC10420681

[fsn371364-bib-0044] Oyaizu, M. 1986. “Studies on Products of Browning Reaction Antioxidative Activities of Products of Browning Reaction Prepared From Glucosamine.” Japanese Journal of Nutrition and Dietetics 44, no. 6: 307–315. 10.5264/eiyogakuzashi.44.307.

[fsn371364-bib-0045] Panjamurthy, K. , S. Manoharan , and C. R. Ramachandran . 2005. “Lipid Peroxidation and Antioxidant Status in Patients With Periodontitis.” Cellular & Molecular Biology Letters 10, no. 2: 255–264 PMID: 16010291. 16010291

[fsn371364-bib-0046] Patel, H. V. , J. D. Patel , and B. Patel . 2014. “Comparative Efficacy of Phytochemical Analysis and Antioxidant Activity of Methanolic Extract of Calotropis Gigantea and *Calotropis procera* .” International Journal of Life Sciences Biotechnology and Pharma Research 5, no. 2: 107–113. 10.2174/2210315508666180608081407.

[fsn371364-bib-0047] Pérez‐Taboada, I. , S. Alberquilla , E. D. Martín , et al. 2020. “Diabetes Causes Dysfunctional Dopamine Neurotransmission Favoring Nigrostriatal Degeneration in Mice.” Movement Disorders 35, no. 9: 1636–1648. 10.1002/mds.28124. 32666590 PMC7818508

[fsn371364-bib-0048] Petersmann, A. , D. Müller‐Wieland , U. A. Müller , et al. 2019. “Definition, Classification and Diagnosis of Diabetes Mellitus.” Experimental and Clinical Endocrinology & Diabetes 127, no. 1: S1–S7. 10.1186/s12906-015-0535-5. 31860923

[fsn371364-bib-0049] Rahmatullah, M. , M. Hosain , S. Rahman , et al. 2013. “Antihyperglycemic and Antinociceptive Activity Evaluation of Methanolic Extract of Whole Plant of *Amaranthus tricolor* L.(Amaranthaceae).” African Journal of Traditional, Complementary, and Alternative Medicines 10, no. 5: 408–411. 10.4314/ajtcam.v10i5.31. PMC3847438 24311858

[fsn371364-bib-0050] Ramachandran, A. 2014. “Know the Signs and Symptoms of Diabetes.” Indian Journal of Medical Research 140, no. 5: 579–581. 25579136 PMC4311308

[fsn371364-bib-0051] Rao, A. A. , G. R. Sridhar , and U. N. Das . 2007. “Elevated Butyrylcholinesterase and Acetylcholinesterase May Predict the Development of Type 2 Diabetes Mellitus and Alzheimer's Disease.” Medical Hypotheses 69, no. 6: 1272–1276. 10.1016/j.mehy.2007.03.032. 17553629

[fsn371364-bib-0052] Rec, G. C. 1972. “Activity of Alkaline Phosphatase.” Journal of Clinical Chemistry and Clinical Biochemistry 10: 182.

[fsn371364-bib-0053] Reitman, S. , and S. Frankel . 1957. “A Colorimetric Method for the Determination of Serum Glutamic Oxalacetic and Glutamic Pyruvic Transaminases.” American Journal of Clinical Pathology 28, no. 1: 56–63. 10.1093/ajcp/28.1.56. 13458125

[fsn371364-bib-0054] Rübsam, A. , S. Parikh , and P. E. Fort . 2018. “Role of Inflammation in Diabetic Retinopathy.” International Journal of Molecular Sciences 19, no. 4: 942. 10.3390/ijms19040942. 29565290 PMC5979417

[fsn371364-bib-0055] Salehi, B. , A. Ata , N. V. Anil Kumar , et al. 2019. “Antidiabetic Potential of Medicinal Plants and Their Active Components.” Biomolecules 9, no. 10: 551. 10.3390/biom9100551. 31575072 PMC6843349

[fsn371364-bib-0056] Searle, P. L. 1984. “The Berthelot or Indophenol Reaction and Its Use in the Analytical Chemistry of Nitrogen. A Review.” Analyst 109, no. 5: 549–568. 10.1039/AN9840900549.

[fsn371364-bib-0057] Secnik, J. , E. Schwertner , M. Alvarsson , et al. 2020. “Cholinesterase Inhibitors in Patients With Diabetes Mellitus and Dementia: An Open‐Cohort Study of Similar to 23 000 Patients From the Swedish Dementia Registry.” BMJ Open Diabetes Research & Care 8, no. 1: 833. 10.1136/bmjdrc-2019-000833. PMC7039592 31958305

[fsn371364-bib-0058] Sen, G. , S. Mandal , S. S. Roy , S. Mukhopadhyay , and T. Biswas . 2005. “Therapeutic Use of Quercetin in the Control of Infection and Anemia Associated With Visceral Leishmaniasis.” Free Radical Biology and Medicine 38, no. 9: 1257–1264. 10.1016/j.freeradbiomed.2005.01.014. 15808423

[fsn371364-bib-0059] Shagirtha, K. , N. Bashir , and S. MiltonPrabu . 2017. “Neuroprotective Efficacy of Hesperetin Against Cadmium Induced Oxidative Stress in the Brain of Rats.” Toxicology and Industrial Health 33, no. 5: 454–468. 10.1177/0748233716665301. 27803291

[fsn371364-bib-0060] Shaker, K. H. , N. Morsy , H. Zinecker , J. F. Imhoff , and B. Schneider . 2010. “Secondary Metabolites From *Calotropis procera* (Aiton).” Phytochemistry Letters 3, no. 4: 212–216. 10.1016/j.phytol.2010.07.009.

[fsn371364-bib-0061] Shewamene, Z. , M. Abdelwuhab , and Z. Birhanu . 2015. “Methanolic Leaf Exctract of *Otostegia integrifolia* Benth Reduces Blood Glucose Levels in Diabetic, Glucose Loaded and Normal Rodents.” BMC Complementary and Alternative Medicine 15, no. 1: 19. 10.1186/s12906-015-0535-5. 25886912 PMC4321712

[fsn371364-bib-0062] Sinha, A. K. 1972. “Colorimetric Assay of Catalase.” Analytical Biochemistry 47, no. 2: 389–394. 10.1016/0003-2697(72)90132-7. 4556490

[fsn371364-bib-0063] Sobeh, M. , M. El‐Raey , S. Rezq , et al. 2019. “Chemical Profiling of Secondary Metabolites of Eugenia Uniflora and Their Antioxidant, Anti‐Inflammatory, Pain Killing and Anti‐Diabetic Activities: A Comprehensive Approach.” Journal of Ethnopharmacology 240: 111939. 10.1016/j.jep.2019.111939. 31095981

[fsn371364-bib-0064] Suryavanshi, S. V. , and Y. A. Kulkarni . 2017. “NF‐κβ: A Potential Target in the Management of Vascular Complications of Diabetes.” Frontiers in Pharmacology 8: 798. 10.3389/fphar.2017.00798. 29163178 PMC5681994

[fsn371364-bib-0065] Suzuki, Y. , and Y. Sakagishi . 1994. “Determination of Serum Bilirubin by the Diazo Method Using the Diazotized 3‐Nitroaniline Reacting Readily With the Photoproducts of Bilirubin.” Japanese Journal of Clinical Chemistry 23, no. 2: 158–163. 10.14921/jscc1971b.23.2_158.

[fsn371364-bib-0066] Thakare, V. , S. S. Shende , P. A. Shirure , and O. C. Swami . 2017. “Role of Conventional Oral Antidiabetic Drugs in Management of Type 2 Diabetes Mellitus.” International Journal of Research in Medical Sciences 5, no. 3: e749. 10.18203/2320-6012.ijrms20170619.

[fsn371364-bib-0067] Thenmozhi, V. , V. Elango , and J. Sadique . 1989. “Anti‐Inflamatory Activity of Some Indian Medicinal Plants.” Ancient Science of Life 8, no. 3&4: 258–261. 22557659 PMC3336731

[fsn371364-bib-0068] Tietz, N. W. 1995. Clinical Guide to Laboratory Tests, 1096. Wiley.

[fsn371364-bib-0069] Tishkowski, K. , and V. Gupta . 2025. “Erythrocyte Sedimentation Rate.” In StatPearls. StatPearls Publishing. 32491417

[fsn371364-bib-0070] Tomic, D. , J. E. Shaw , and D. J. Magliano . 2022. “The Burden and Risks of Emerging Complications of Diabetes Mellitus.” Nature Reviews Endocrinology 18, no. 9: 525–539. 10.1038/s41574-022-00690-7. PMC9169030 35668219

[fsn371364-bib-0071] Tsado, N. A. , B. Lawal , P. C. Ossa , et al. 2016. “Antioxidants and Antimicrobial Activities of Methanol Extract of Newbouldia Laevis and Crateva Adansonii.” Journal of Pharmacy and Allied Health Sciences 6, no. 1–2: 14–19. 10.3923/jpahs.2016.14.19.

[fsn371364-bib-0072] Vaknine, S. , and H. Soreq . 2020. “Central and Peripheral Anti‐Inflammatory Effects of Acetylcholinesterase Inhibitors.” Neuropharmacology 168: 108020. 10.1016/j.neuropharm.2020.108020. 32143069

[fsn371364-bib-0073] Van Handel, E. , and D. B. Zilversmit . 1957. “Micromethod for the Direct Determination of Serum Triglycerides.” Journal of Laboratory and Clinical Medicine 50, no. 1: 152–157. 13439279

[fsn371364-bib-0074] Yadav, C. K. , A. Chaube , S. Thapa , A. Palikhey , L. Shrestha , and K. Kandel . 2023. “In‐Vitro Anti‐Diabetic Activity and Phytochemical Screening of Ethanolic Extract of *calotropis gigantea* (Linn).” Journal of Universal College of Medical Sciences 11, no. 2: 50–53. 10.3126/jucms.v11i02.58064.

[fsn371364-bib-0075] Yaribeygi, H. , T. Sathyapalan , S. L. Atkin , and A. Sahebkar . 2020. “Molecular Mechanisms Linking Oxidative Stress and Diabetes Mellitus.” Oxidative Medicine and Cellular Longevity 2020, no. 1: 8609213. 10.1155/2020/8609213. 32215179 PMC7085395

[fsn371364-bib-0076] Yusuf, A. A. , B. Lawal , U. B. Alozieuwa , et al. 2023. “Attenuating Effects of *Azanza garckeana* Fractions on Glycemo‐Impaired‐Associated Dyslipidemia, Hepatopathy, and Nephropathy.” American Journal of Translational Research 15, no. 10: 5997. 10.1016/j.biopha.2022.113196. 37969197 PMC10641334

[fsn371364-bib-0077] Zhou, B. , A. W. Rayner , E. W. Gregg , et al. 2024. “Worldwide Trends in Diabetes Prevalence and Treatment From 1990 to 2022: A Pooled Analysis of 1108 Population‐Representative Studies With 141 Million Participants.” Lancet 404, no. 10467: 2077–2093. 10.1016/s0140-6736(24)02317-1. 39549716 PMC7616842

[fsn371364-bib-0078] Zhou, B. , H. Zou , and G. Xu . 2016. “Clinical Utility of Serum Cystatin c in Predicting Diabetic Nephropathy Among Patients With Diabetes Mellitus: A Meta‐Analysis.” Kidney and Blood Pressure Research 41, no. 6: 919–928. 10.1159/000452593. 27889768

